# Contemporary Theory and Research

**Published:** 1893-07-01

**Authors:** 


### CONTEMPORARY THEORY AND RESEARCH.

### A PINCH OF DUST

The dangers that lurk unseen in the air form the subject of an essay by M. de Nansouty on "The Atmosphere of Large Towns and Micrography." He points out the increased pollution of the air in Paris from the factories worked by steam machinery, and remarks that vapours which contain sulphur are specially disastrous to the lungs, since the sulphur which they contain is transformed into sulphurous acid, and then into sulphuric acid which falls back to the earth with the rain and fog. An analysis of dust particles reveals that a remarkable collection of diverse objects may be absorbed at every breath in the street of a large city; silex, chalk, plaster, pulverised rock, charcoal, hairs, fibres, vegetable refuse, starch, pollen cells, &c. A specimen of dust collected from furniture on the third floor of a street in Rennes contained all this and nearly three million bacteria in addition. A gramme of dust (about 15 grains) in movement in the streets encloses about 130,000 bacteria. The dust, of houses, then is far more dangerous. M. de Nansouty concludes that it is of incalculable importance to devote incessant attention to the number, quality, and nature of these microscopic beings which surround us.

### THE CONTAGIOUSNESS OF CONSUMPTION.

Both in France and America the risks incurred by the lack of antiseptic precautions in consumptive cases are receiving serious attention. Dr. Hopkins, of Thomasville, in stating that he has joined the growing army which placed tuberculosis in the category of contagious diseases explains that his experience for nineteen years with this disease in a consumptive resort has made him a willing subject of Koch. He does not doubt that all persons receive the tubercle bacilli at some time or other into their air passages, but fortunately the great majority possess the power of repelling them. Indians in a state of nativity seem impervious to the germs of consumption, but are now dying by thousands on the reservations. The whites and the blacks in prisons all the world over labour under similar conditions. A report from the Illinois State Prison at Joliet says that there are 1,400 convicts, and that fully one-third of them have consumption in a light or bad form; nearly all deaths of persons in the penitentiary have been caused by consumption. Dr. Hopkins emphasises the danger that lurks in sleeping cars in carpets, bedding, clothing, and in the walls of apartments occupied by consumptives which have not been properly disinfected. He considers that the time may be approaching when resorts now soliciting the patronage of the consumptive will be quarantining against him. In Paris, it appears, the hospitals are increasingly over-charged with phthisical patients, so much so that the Society of Medicine and Professional Hygiene has lately issued a request for the founding of a special hospital devoted to the treatment of this disease "which contaminates the convalescents and ordinary patients of the general hospitals."

### EPILEPSY IN THE SOUDAN.

Dr. Rançon, of the French navy, has published some interesting observations in the "Archives de Médecine Navale" on this subject. He writes: "Epilepsy is far from being as rare as one might imagine among the negroes of the Soudan. It is one of those complaints which they hide, and, indeed, if you merely pass through a village and pay a short visit you will see no one either infirm or ill. The blind alone have free liberty to wander about. But others, the insane, lepers, epileptics, are confined in their huts, and mix rarely with the others. But if you stay several months in one village, little by little the natives grow familiar with you and let you see corners of their life which otherwise would always remain unknown to you. From what we have been able to see and observe, the background of a negro village is not exactly beautiful, speaking always, be it understood, from a pathological point of view. All the maladies engendered by insufficient nourishment and profound physiological poverty are here represented: pulmonary phthisis, leprosy, elephantiasis abound. Of all nervous diseases epilepsy is, perhaps, that which is the most wide-spread and common. There is hardly a village which has not at least one and often two or more epileptics. In each country the blacks have a particular name to designate this appalling malady, while for most other diseases they have none. This is, perhaps, the most striking proof of the impression made on the Soudanese by epilepsy. The unhappy victims who are attacked by it are regarded in the villages as mad; no care is taken of them; they are not even watched. But neither are they ill-treated, and whatever harm they do is passed over as the action of an irresponsible being. It may easily be understood after this that the unhappy epileptics mostly come to a tragic end. They are either drowned or burnt during a fit or kill themselves by falling from trees, &c. Hardly one dies a natural death."

